# The mechanoreceptor *pezo-1* is required for normal crawling locomotion in the nematode *C. elegans*

**DOI:** 10.17912/micropub.biology.001085

**Published:** 2023-12-19

**Authors:** Adithya Komandur, Adina Fazyl, Wolfgang Stein, Andrés G. Vidal-Gadea

**Affiliations:** 1 Normal Community High School, Normal, Illinois, United States; 2 School of Biological Sciences, Illinois State University, Normal, Illinois, United States

## Abstract

The discovery in 2010 of the PIEZO family of mechanoreceptors revolutionized our understanding of the role of proprioceptive feedback in mammalian physiology. Much remains to be elucidated. This study looks at the role this receptor plays in normal locomotion. Like humans, the nematode *C. elegans* expresses PIEZO-type channels (encoded by the *pezo-1* gene) throughout its somatic musculature. Here we use the unbiased automated behavioral software Tierpsy to characterize the effects that mutations removing PEZO-1 from body wall musculature have on *C. elegans* crawling. We find that loss of PEZO-1 results in disrupted locomotion and posture, consistent with phenotypes associated with loss of PIEZO2 in human musculature. *C. elegans* is thus an amenable system to study the role of mechanoreception on muscle physiology and function.

**Figure 1. Loss of mechanoreceptor pezo-1 alters the crawling behavior of C. elegans:**
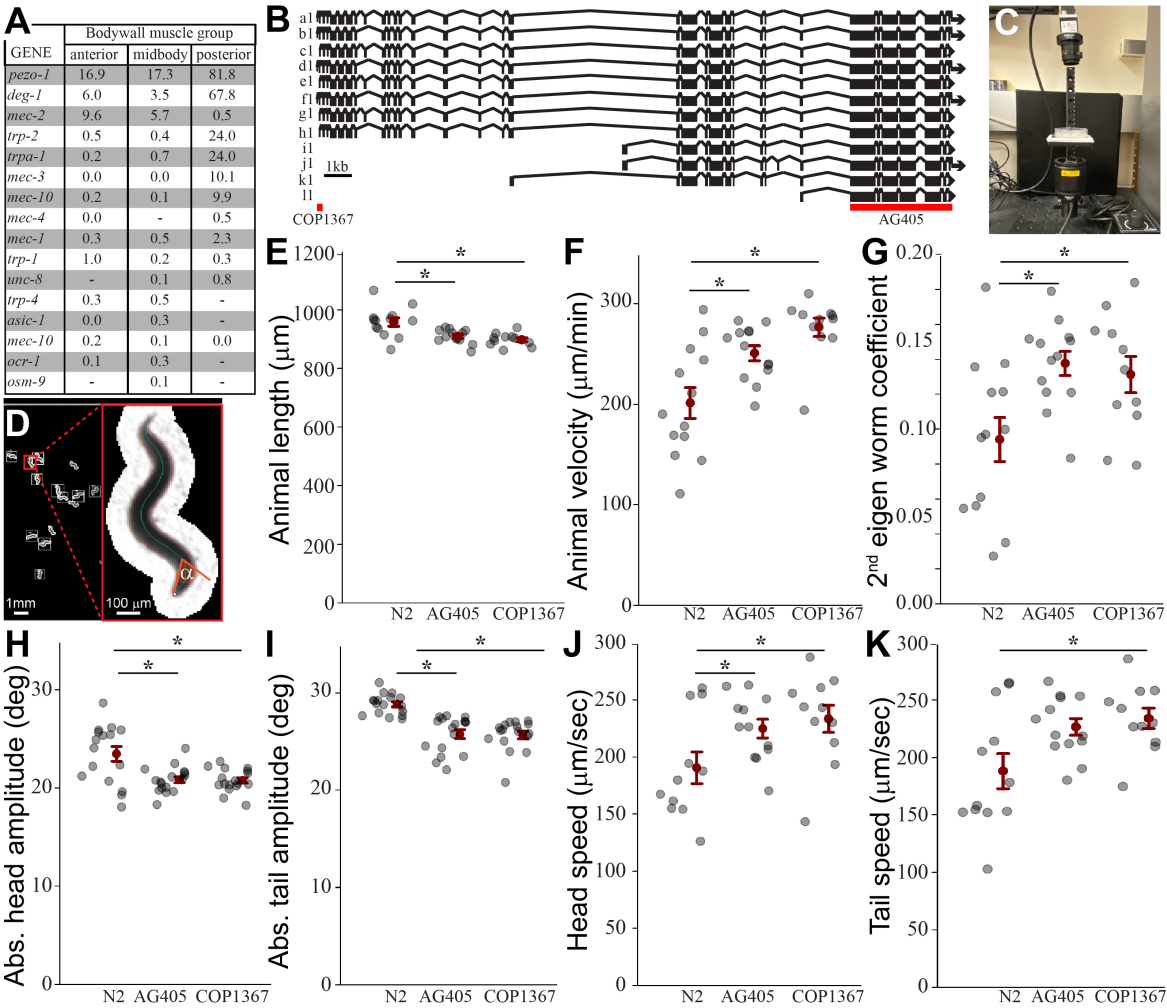
**A)** Table showing Wormseq transcriptomic data for all known mechanoreceptors with expression in body wall muscles. *pezo-1* is the mechanoreceptor with the greatest expression in adult *C. elegans* . **B)** The mechanoreceptor *pezo-1* in *C. elegans* is comprised of a dozen isoforms. Diagram (adapted from Hughes et al., 2021) showing the alignment between different *pezo-1* isoforms. Red bars identify the CRISPR-introduced mutations for the strains used in this study. COP1367 impairs the production of long *pezo-1* isoforms expressed in body wall musculature. AG405 is characterized by a long deletion in the shared C-terminus of the gene which results in a null mutant for all *pezo-1* isoforms. **C)** Filming setup incorporating an infrared camera and infrared LED light source. **D)** Example data analysis from the behavioral tracking software TIERPSY, showing the tracking of multiple worms in exquisite detail. **E)** Comparison of mean animal length between wild-type worms ( N2 ) and *pezo-1* knock-out mutants. **F)** Comparison of mean animal speed of wild-type worms and *pezo-1* mutants. **G)** Second eigen worm coefficient for wildtype and *pezo-1* mutants. **H)** Average absolute head amplitude for crawling wildtype and *pezo-1* mutant worms. **I)** Average absolute tail amplitude for crawling wildtype and *pezo-1* mutant worms. **J)** Average head (and tail: **K** ) speed for crawling wildtype and *pezo-1* mutant worms.

## Description

Proprioception is crucial for the production of coordinated locomotion. In mammals, proprioceptive organs such as Golgi tendon organs and muscle spindles provide the nervous system with continuous feedback about muscle tension and length respectively, and mediate compensatory reflex loops (Moore et al., 1984; Kröger et al., 2021).

For decades, the identity of the mechanoreceptor proteins mediating most proprioceptive feedback in mammals remained elusive. This changed just a decade ago with the discovery of the Piezo family (Piezo1 and Piezo2) of mechanosensitive ion channels and led to a watershed moment in our understanding of mammalian mechanoreception (Volkers et al., 2015) . We now know that PIEZO receptors are integral parts of both Golgi and muscle spindle organs (Haliloglu et al., 2017) .

While Golgi organs and muscle spindles monitor whole muscle length and tension, muscles themselves are comprised of hundreds of thousands of individual muscle cells. For example, the human biceps comprises a quarter million individual muscle cells (Klein et al., 2003) . Recent evidence suggests that mechanoreceptors like PIEZO and TRP channels are not only expressed in proprioceptive organs but also in individual muscle cells (Haliloglu et al., 2017) . Since these mechanoreceptors are not associated with the nervous system and hence do not provide proprioceptive feedback, it remains undetermined what the role of these muscle-bound receptor channels is in muscle function.

The nematode *C. elegans* has an ortholog of mammalian PIEZO channels ( *pezo-1* ). We used the transcriptome library Wormseq (Ghadar et al., 2023) to search for mechanoreceptor genes with known expression in *C. elegans* body wall musculature. Of 16 identified mechanoreceptor genes with body-wall expression in adult worms, *pezo-1* was the one with the greatest level of expression across these muscles ( Figure 1A ). We therefore determined that this mechanoreceptor was an optimal choice to investigate the role of mechanoreceptor expression in muscle function.

Like human PIEZO channels, *C. elegans* has about a dozen isoforms with differential expression patterns ( Figure 1B ; Hughes et al., 2022; Millet et al., 2021; Bai et al., 2020). The role that *pezo-1* , or other mechanoreceptors, play in skeletal muscle function remains undetermined.

We performed an in-depth unbiased analysis of *C. elegans* locomotion in control ( N2 ) and *pezo-1* mutant animals to establish what alterations (if any) result from the loss of *pezo-1* in nematode skeletal muscles. We found that loss of PEZO-1 resulted in several significant alterations to nematode crawling behavior which included decreased angular excursion and increased forward velocity.

We used wild-type worms ( N2 ) and two *pezo-1* knockout lines. Hughes et al (2022) previously reported expression of the longest isoforms (a-h) in the worms’ body wall muscles. We therefore used the strains COP1367 (kind gift of Dr. Vásquez) which contains a knock-out of long *pezo-1* isoforms expressed in muscles. Additionally, we used the AG405 strain, which is missing all *pezo-1* isoforms (kind gift of Dr. Goldman and Bai). Groups of day-1 gravid hermaphrodite worms were filmed using an infrared light/camera system ( Figure 1C ). We filmed between 13 and 17 assays per condition with about 20-40 worms per assay. Videos were then analyzed using Tierpsy, an open-source platform for analyzing worm behavior data ( Figure 1D ). As an initial screening step, we used one-way ANOVAs to identify variables that showed significant differences (alpha level = 0.05) between control animals and both *pezo-1* mutants. Out of thousands of possible kinematic parameters, we identified over 200 as being significantly altered in both *pezo-1* mutants compared to N2 controls. *pezo-1* mutants were slightly shorter than wildtypes ( Figure 1E ). We found that *pezo-1* mutants crawled faster than controls ( Figure 1F ). This faster speed did not seem to result from simply sped-up kinematics but it involved the differential use of moving strategies. This is supported by changes in eigen vector (eigen worm) coefficients ( Figure 1G ) where *pezo-1* mutants are seen engaging significantly more in this and other motor patterns than wild types (Stephens et al., 2008) . We note that rather than engaging in a different motor program, these mutants seem to move within the normal range of locomotion but cluster towards the higher end of the normal (control) range. This is also seen in measurements of the head ( Figure 1H ), and tail ( Figure 1I ) absolute amplitude during crawling locomotion where *pezo-1* mutants clustered towards the bottom of the normal ( N2 ) range. This decrease in movement amplitude was accompanied by an increase in head ( Figure 1J ) and tail ( Figure 1K ) velocities.

Together these data suggest that loss of *pezo-1* impacts body-wall muscle activity during crawling in *C. elegans* . Worms crawl by generating alternating dorsoventral body-wall contractions. The observation that *pezo-1* mutants display both a decrease in body bend amplitude and an increase in forward velocity suggests that loss of *pezo-1* in body-wall muscles leads to increased muscle activation and more muscle force. This would be consistent with loss of PIEZO mutations in human patients, which have been associated with muscle stiffness and decreased range of motion (Ma et al., 2019) . Understanding how mechanoreceptors like *pezo-1* mediate normal muscle function and adaptability remains an understudied field with the potential to impact our knowledge of basic muscle physiology as well as the many conditions that can challenge muscle performance and viability.

## Methods

Animal husbandry. Worms were grown in standard nematode growth media (NGM) plates and fed on *E.coli* strain OP50 at 20°C until they reached day one of adulthood.

Behavioral Assays. Twenty to forty gravid day-one adult hermaphrodite worms (24 hrs post mid L4 stage at 20°C) were transferred to the center of a 5cm diameter NGM plate devoid of food and placed in a behavioral rig consisting of a Basler infrared camera (ace CMOS, acA4024-29um) and illuminated by an infrared light source (M850L3-C1 850nm, 480mW, 1200mA LED, Figure 1C ). Animals were allowed to acclimatize for five minutes and then they were filmed freely crawling at 30fps and at a resolution of 1920x1080 pixels in a 4cm radius arena for 20 seconds. We filmed a minimum of 13 assays for each of the three strains.

Behavioral Analysis. We used the Tierpsy Tracker behavioral package (version 1.5.1-beta2) to analyze the movies and generate a list of kinematic metrics describing each strain (Javer et al., 2018) . Each video was hand-curated to ensure the software correctly identified and measured real worms. Tierpsy analysis then produces a matrix summarizing over 4,000 kinematic measurements for each assay population. We used one-way ANOVAs to compare measurements of N2 wild types and the two *pezo-1* mutant strains. To be conservative we only focused on parameters where both *pezo-1* strains were statistically different from wildtypes (>200 metrics). Using this list, we identified families of parameters that reflected differences between similar metrics between mutant and wild-type strains.

Statistical analysis. We used Sigmaplot 14.1 to run comparisons between different groups. Parametric data displaying similar variance was compared using one-way ANOVAs which were followed up by Dunnett's test using N2 as controls. *P<0.05.

## Reagents

N2 strains were provided by the CGC, which is funded by the NIH Office of Research Infrastructure Programs (P40 OD010440). The COP1553 strain was a kind gift from Dr. Valeria Vásquez. The AG405 strain was a kind gift from Drs. Andy Golden and Xiaofei Bai.

Table 1. Nematode strains used in this study.

**Table:** 

**Strain**	**Allele**	**Position**	**Mutation/genotype**	**Notes**
N2	n/a	n/a	WT	Reference strain
COP1367	*knu384*	IV:9366441	*pezo-1 ( knu384 )* - 198bp deletion and 23bp insertion: gaatcggtggtcgtaacacagcaacaacaga//ATG ACGGTCCCGCC - deletion of 198bp and insertion of ACAGCAACAACAGAATGACGGTC - TCGAAAGTCTTTAGCACTTCCCAgtaaga aattaaagtccgatgt	leads to premature stop - MTVPPQQQQNDGLESL*
AG405	*av143*	unknown	*pezo-1 ( av143 )* - C-terminus deletion of Exon 27-33 + introns, generated by CRISPR/Cas9, Inserted STOP CODON AFTER deletion	Deletion affecting all isoforms

## References

[R1] Bai X, Bouffard J, Lord A, Brugman K, Sternberg PW, Cram EJ, Golden A (2020). Caenorhabditis elegans PIEZO channel coordinates multiple reproductive tissues to govern ovulation.. Elife.

[R2] Ghaddar A, Armingol E, Huynh C, Gevirtzman L, Lewis NE, Waterston R, O'Rourke EJ (2023). Whole-body gene expression atlas of an adult metazoan.. Sci Adv.

[R3] Haliloglu G, Becker K, Temucin C, Talim B, Küçükşahin N, Pergande M, Motameny S, Nürnberg P, Aydingoz U, Topaloglu H, Cirak S (2016). Recessive PIEZO2 stop mutation causes distal arthrogryposis with distal muscle weakness, scoliosis and proprioception defects.. J Hum Genet.

[R4] Hughes K, Shah A, Bai X, Adams J, Bauer R, Jackson J, Harris E, Ficca A, Freebairn P, Mohammed S, Fernández EM, Bainbridge C, Brocco M, Stein W, Vidal-Gadea AG (2022). Distinct mechanoreceptor pezo-1 isoforms modulate food intake in the nematode Caenorhabditis elegans.. G3 (Bethesda).

[R5] Klein CS, Marsh GD, Petrella RJ, Rice CL (2003). Muscle fiber number in the biceps brachii muscle of young and old men.. Muscle Nerve.

[R6] Kröger S, Watkins B (2021). Muscle spindle function in healthy and diseased muscle.. Skelet Muscle.

[R7] Javer A, Ripoll-Sánchez L, Brown AEX (2018). Powerful and interpretable behavioural features for quantitative phenotyping of Caenorhabditis elegans.. Philos Trans R Soc Lond B Biol Sci.

[R8] Ma Y, Zhao Y, Cai Z, Hao X (2019). Mutations in PIEZO2 contribute to Gordon syndrome, Marden-Walker syndrome and distal arthrogryposis: A bioinformatics analysis of mechanisms.. Exp Ther Med.

[R9] Millet JRM, Romero LO, Lee J, Bell B, Vásquez V (2021). C. elegans PEZO-1 is a mechanosensitive ion channel involved in food sensation.. J Gen Physiol.

[R10] Moore JC (1984). The Golgi tendon organ: a review and update.. Am J Occup Ther.

[R11] Stephens GJ, Johnson-Kerner B, Bialek W, Ryu WS (2008). Dimensionality and dynamics in the behavior of C. elegans.. PLoS Comput Biol.

[R12] Volkers L, Mechioukhi Y, Coste B (2014). Piezo channels: from structure to function.. Pflugers Arch.

