# Corrigendum: The mechanoreceptor *pezo-1* is required for normal crawling locomotion in the nematode *C. elegans*

**DOI:** 10.17912/micropub.biology.001723

**Published:** 2025-06-25

## Description

The authors requested a correction to the Funding section of this article. The text now reads: 

"This research was funded by the National Science Foundation (NSF-1818140) and the National Institutes of Health (NIH 2R15AR068583-02). The NIH R15 award supported infrastructure development and student participation integral to the successful execution of this project. "

The text originally read: 

"Supported by National Science Foundation (United States) 1818140 to Andres Vidal-Gadea"

